# Deciphering the Role of ERBB3 Isoforms in Renal Cell Carcinoma: A Comprehensive Genomic and Transcriptomic Analysis

**DOI:** 10.3390/medicina60010181

**Published:** 2024-01-20

**Authors:** Mingyu Kim, Hyung Ho Lee, So Dam Won, YeonSue Jang, Baek Gil Kim, Nam Hoon Cho, Young Deuk Choi, Jin Soo Chung, Hyun Ho Han

**Affiliations:** 1 Center for Urologic Cancer, National Cancer Center, 323, Ilsan-Ro, Ilsandong-Gu, Goyang-Si 10408, Gyeonggi-Do, Republic of Korea; kmk63819@ncc.re.kr (M.K.); uroh@ncc.re.kr (H.H.L.); wonsodam1112@gmail.com (S.D.W.); 2 Department of Pathology, Yonsei University College of Medicine, Seoul 03722, Republic of Korea; ysjang@yuhs.ac (Y.J.); bbaekiri@yuhs.ac (B.G.K.);; 3 Department of Urology, Urological Science Institute, Yonsei University College of Medicine, 50-1, Yonsei-Ro, Seodaemun-Gu, Seoul 03722, Republic of Korea; youngd74@yuhs.ac

**Keywords:** ERBB3 isoforms, renal cell carcinoma, TCGA

## Abstract

ERBB3, a key member of the receptor tyrosine kinase family, is implicated in the progression and development of various human cancers, affecting cellular proliferation and survival. This study investigated the expression of ERBB3 isoforms in renal clear cell carcinoma (RCC), utilizing data from 538 patients from The Cancer Genome Atlas (TCGA) Firehose Legacy dataset. Employing the SUPPA2 tool, the activity of 10 ERBB3 isoforms was examined, revealing distinct expression patterns in RCC. Isoforms uc001sjg.3 and uc001sjh.3 were found to have reduced activity in tumor tissues, while uc010sqb.2 and uc001sjl.3 demonstrated increased activity. These variations in isoform expression correlate with patient survival and tumor aggressiveness, indicating their complex role in RCC. The study, further, utilizes CIBERSORTx to analyze the association between ERBB3 isoforms and immune cell profiles in the tumor microenvironment. Concurrently, Gene Set Enrichment Analysis (GSEA) was applied, establishing a strong link between elevated levels of ERBB3 isoforms and critical oncogenic pathways, including DNA repair and androgen response. RT-PCR analysis targeting the exon 21–23 and exon 23 regions of ERBB3 confirmed its heightened expression in tumor tissues, underscoring the significance of alternative splicing and exon utilization in cancer development. These findings elucidate the diverse impacts of ERBB3 isoforms on RCC, suggesting their potential as diagnostic markers and therapeutic targets. This study emphasizes the need for further exploration into the specific roles of these isoforms, which could inform more personalized and effective treatment modalities for renal clear cell carcinoma.

## 1. Introduction

Renal cell carcinoma (RCC) is a type of kidney cancer that originates in the lining of the proximal convoluted tubule, a part of the very small tubes in the kidney that transport primary urine. RCC is the most common type of kidney cancer in adults, responsible for approximately 90–95% of cases [1,2,3,4]. Due to the complex nature of RCC, there are many cases where the correct treatment timing is missed, and various biomarkers are being studied to resolve this problem and improve treatment effectiveness through the early diagnosis and confirmation of the prognosis of the disease [5,6,7,8]. The ErbB family of receptor tyrosine kinases, crucial for cell growth, survival, and differentiation, can lead to cancer when dysregulated. Specifically, the overexpression of ErbB receptors activates pathways like PI3K/Akt and MAPK, promoting cancer progression. ERBB3 (HER3), a unique member of this family, requires dimerization with other receptors like ERBB2 (HER2) for signaling. This is particularly significant in kidney disease, where ERBB3’s role in key signaling pathways influences cell survival and proliferation, contributing to tumor growth [9,10,11]. Significantly, ERBB3 isoforms have received attention in various studies. Isoforms, which are variants of the same protein, often differ in their biological activity and interactions within cellular pathways. The expression patterns and functions of these ERBB3 isoforms can vary greatly, potentially impacting the tumor behavior, response to therapy, and interaction with the tumor microenvironment [12,13,14,15,16]. Understanding the distinct roles and mechanisms of these isoforms is imperative, as it opens new possibilities for targeted therapeutic interventions. The ongoing research into ERBB3 isoforms in RCC not only helps us to learn more about the disease’s molecular biology, but also shows that targeting specific isoforms could be a new way to treat RCC.

## 2. Materials and Methods

### 2.1. Medical Samples

For this investigation, normal and tumor tissue samples were taken from a total of 26 RCC (renal cell carcinoma) patients. After obtaining written informed consent and the study being reviewed by the National Cancer Center Institutional Review Board (IRB no: NCC2021-0147), patient tissue samples were collected. The clinical information of the subjects is summarized in Table 1.

### 2.2. Examining the UCSC Cancer Genomics Browser

A crucial online resource that provides both public and private access to multi-omics functional genomics and clinical/phenotypic datasets is the UCSC Genome Browser (https://genome.ucsc.edu/) (Accessed: 20 December 2023) [17,18,19]. In this work, we used the UCSC Genome Browser on the human assembly GRCh37/hg19 to identify 10 different ERBB3 isoforms.

### 2.3. Measurement of ERBB3 Isoforms Using SUPPA2

We utilized SUPPA2 to extract and quantify alternative splicing (AS) events from TCGA and GTEx samples [20]. Reads were mapped using STAR with the quantMode PTranscriptomeSAM option so that alternative splicing could be analyzed and isoforms could be counted. This approach is efficient for RNA-seq data, providing accurate transcriptome mapping. Following this, isoform quantification was conducted using Salmon (version 1.2.1) [21], a robust tool for transcript expression quantification, known for its accuracy and efficiency. Differential gene expression analysis was carried out using the DESeq2 package in the R environment [22,23,24]. DESeq2 is widely used for such analyses due to its statistical approach that accounts for variance and size factors in RNA-seq data, making it highly suitable for differential expression analysis.

### 2.4. Isoform Analysis of ERBB3 Making Use of the cBioPortal Database

Using the cBioPortal database (https://www.cbioportal.org/) (Accessed: 20 December 2023), we retrieved 538 patient records from the “Kidney Renal Clear Cell Carcinoma (TCGA, Firehose Legacy)” dataset. This website provides data on gene expression patterns, sickness prognosis, and patient survival rates [25]. Using the available data, we used SUPPA2 to identify and depict the link between ERBB3 isoform expression levels and a variety of clinical variables, such as tumor size, cancer aggressiveness and stage, and patient survival rate.

### 2.5. Analysis of CIBERSORT Deconvolution

Based on gene expression data, the relative proportions of various cell types inside the tumor microenvironment were inferred using CIBERSORT, a computational method that uses a deconvolution algorithm [26]. The fractions of 22 human hematopoietic cell phenotypes were estimated using a leukocyte gene signature matrix (LM22). 

### 2.6. Gene Set Enrichment

Hallmark gene sets are groups of genes that contain certain, well-defined biological states or processes and demonstrate consistent expression. These gene sets were obtained from the MsigDB [27], which is a reference database. In addition, the C2 curated gene sets were acquired for the purpose of conducting more research. These sets combine the online route databases with the publications in the field of biomedicine. For validating the data that were acquired and the levels of isoform expression for each individual patient, the Spearman’s rank correlation coefficient was also used. Following this, a heatmap was created by using GraphPad Prism version 10.1.0 to display the correlation values for every isoform found.

### 2.7. Reverse Transcription Polymerase Chain Reaction (RT-PCR)

To analyze mRNA expression and detect ERBB3 mutations in renal cell carcinoma (RCC) and healthy kidney tissues, total RNA was extracted using TRIzol Reagent (Invitrogen, Bartlesville, OK, USA). For reverse transcription, 1 μg of RNA was combined with PrimeScript RT Master Mix (Takara Bio Inc., Kusatsu, Shiga, Japan) and incubated at 37 °C for 15 min. PCR amplification involved denaturation at 95 °C for 20 s, annealing at 54 °C for 18S gene, 57 °C for 23_exon gene, and 55 °C for 21_exon gene, each for 40 s, and extension at 72 °C for 1 min. The PCR products were run on a 2% agarose gel and stained with a nucleic acid solution (iNtRON, Jungwon-gu, Seongnam-si, Korea). Primer sequences are detailed in Table 2.

### 2.8. Statistical Analysis

The statistical analysis of all experimental outcomes was represented using the mean ± standard deviation (mean ± SD). To assess the statistical significance between each group, a one-way *t*-test was conducted using GraphPad Prism software version 10.1.0 (GraphPad Software, La Jolla, CA, USA). Statistical significance was deemed to be present only when the *p*-values were less than 0.05, 0.01, and 0.001, respectively, in comparison to the results obtained via Tukey’s multiple comparison test.

## 3. Results

### 3.1. Investigation of ERBB3 Isoform Expression in RCC Using SUPPA2

**Figure 1:**
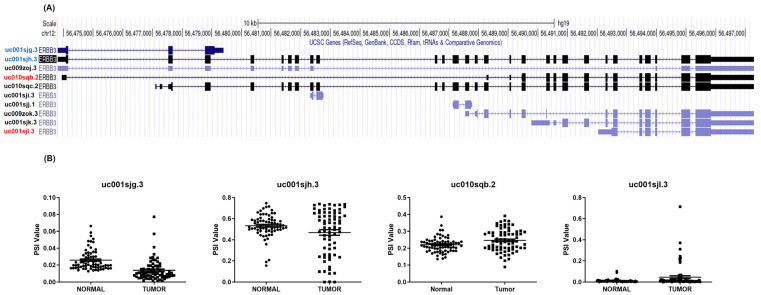
Comparative analysis of ERBB3 isoform expression in normal and tumor tissues of renal clear cell carcinoma patients. (**A**) ERBB3 isoform analysis in renal clear cell carcinoma from TCGA Firehose Legacy Dataset overview of the 10 ERBB3 isoforms identified in the UCSC Genome Browser, evaluated using the SUPPA2 tool. The analysis includes data from 538 individuals with renal clear cell carcinoma, focusing on the initial dataset and the specific ERBB3 isoforms under investigation. (**B**) The differential expression of ERBB3 isoforms in normal vs. tumor tissues shows how the expression levels of ERBB3 isoforms change in renal clear cell carcinoma patients’ normal tissues compared to their tumor tissues. It specifically contrasts the expression patterns of uc001sjg.3 and uc001sjh.3 (reduced expression in tumor tissues) against uc010sqb.2 and uc001sjl.3 (increased expression in tumor tissues).

Data from 538 RCC patients from the TCGA Firehose Legacy dataset were analyzed. The study identified 10 ERBB3 isoforms using the SUPPA2 tool and the UCSC Genome Browser. It was found that isoforms uc001sjg.3 and uc001sjh.3 had reduced expression in tumor tissues, while uc010sqb.2 and uc001sjl.3 were more expressed in tumors (Figure 1A,B). This indicates the diverse roles of ERBB3 isoforms in RCC.

### 3.2. Comparative Analysis of uc001sjh.3 and uc001sjl.3 Isoforms Regarding Cancer Prognosis and Patient Survival

**Figure 2:**
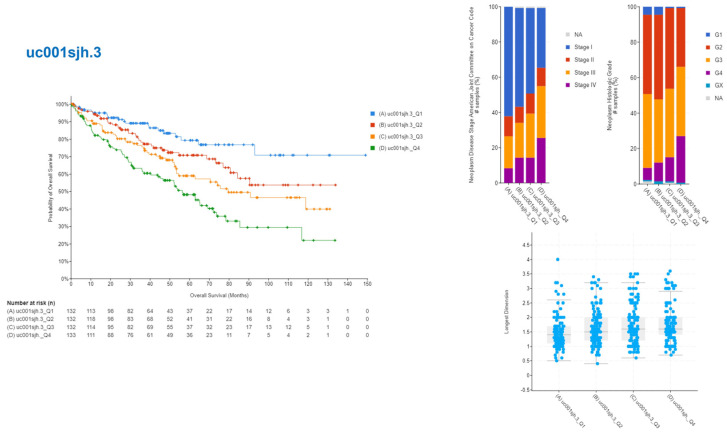
The impact of uc001sjh.3 isoform expression on patient survival and tumor characteristics. Illustration of the correlation between the expression level of the uc001sjh.3 isoform and both the patient’s lifespan and the characteristics of tumors in malignant tissues. The graph illustrates a significant correlation between elevated levels of uc001sjh.3 expression (in the top quartile, Q1) and increased patient survival rates. Over 70% of the patients achieved a survival rate exceeding 150 months. Moreover, it demonstrates that malignancies exhibiting elevated levels of uc001sjh.3 expression exhibit a slower growth rate and smaller tumor size. The data points are classified into quartiles, where Q1 denotes the highest levels of expression. To demonstrate these associations, we present the survival curve, tumor size distribution, and malignancy advancement rate. The blue dots in the picture showing tumor size represent each patient.

**Figure 3:**
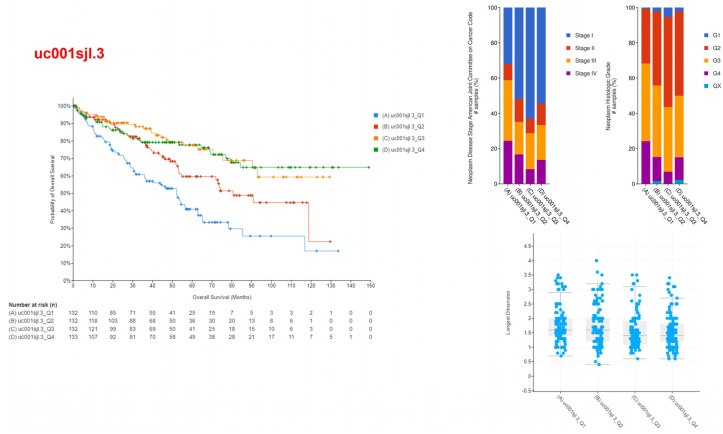
The correlation of uc001sjl.3 isoform expression with decreased survival rates and tumor aggressiveness illustrates the correlation between elevated levels of the uc001sjl.3 isoform and both patient survival and the aggressiveness of tumors in malignant tissues. Evidence demonstrates that increased levels of uc001sjl.3 (upper quartile, Q1) expression are associated with considerably reduced survival rates, especially when less than 20% of patients survive beyond 130 months. The image also illustrates the correlation between elevated uc001sjl.3 expression and heightened tumor aggressiveness and size. The data are partitioned into quartiles, with Q1 denoting the greatest levels of expression. These findings are visually represented using a survival curve, as well as graphs illustrating tumor size and aggressiveness. The blue dots in the picture showing tumor size represent each patient.

The upper quartile, representing the top 25% of individuals, was labeled as Q1, while the lower quartile, representing the lowest 25% of individuals, was labeled as Q4, based on the analysis of gene expression levels. The connection between the expression levels of these isoforms and patients’ illness prognosis and survival rates was examined using cBioportal. Two of the identified genes, uc001sjh.3 and uc001sjl.3, had notable associations. An elevated expression level of uc001sjh.3 showed a significant correlation with enhanced patient survival rates, as more than 70% of patients demonstrated survival beyond a span of 150 months. It was also found that tumors that had high levels of uc001sjh.3 gene expression tended to be less malignant and spread less quickly, and they were also smaller (Figure 2). On the other hand, the transcript uc001sjl.3 demonstrated contrasting outcomes, as increased levels of expression were shown to be associated with decreased survival rates. Specifically, the Q1 group displayed survival rates of fewer than 20% at 130 months. Bigger tumors were present in cancers with higher levels of uc001sjl.3 expression, indicating greater aggressiveness and advancement (Figure 3). Additionally, an examination was conducted into the potential link between the survival rate of patients and the two remaining isoforms, uc001sjg.3 and uc010sqb.2. However, no statistically significant association was observed between the expression level of these genes and the survival rate (Figure S2).

### 3.3. CIBERSORTx and Gene Set Enrichment Analysis (GSEA) to Elucidate the Relationship between ERBB3 Isoforms and Immune Cell Profiles in Cancer Progression

**Figure 4:**
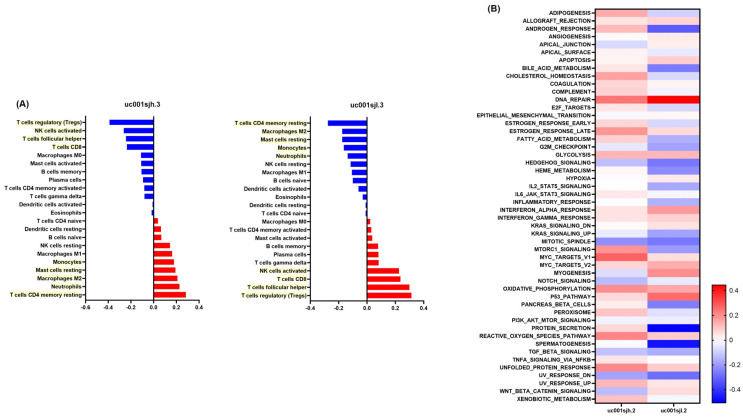
Exploring ERBB3 isoform expression and its impact on immune cell profiles and gene set enrichment in renal cell carcinoma. (**A**) CIBERSORTx analysis exploring the relationship between the expression levels of ERBB3 isoforms (uc001sjh.3 and uc001sjl.3) and the proportional representation of immune cells in the cancer samples. The deconvolution values from CIBERSORTx, confirmed via Spearman correlation analysis, revealed distinct immune cell profiles associated with each isoform. For uc001sjh.3, a negative association was noted with regulatory T cells (Tregs), activated NK cells, follicular helper T cells, and CD8 T cells, while a positive correlation was seen with CD4 memory resting T cells, neutrophils, M2 macrophages, resting mast cells, and monocytes. In contrast, uc001sjl.3 showed inverse relationships, indicating its potential role in diminishing innate immunity, which could contribute to increased cancer growth and aggressiveness. Nine different types of immune cells, each exhibiting varying relationships with the two isoforms, are highlighted in yellow to emphasize these correlations. (**B**) Gene set enrichment analysis (GSEA) explores the association between the overexpression of ERBB3 isoforms and the enrichment of specific gene sets in renal cell carcinoma. Fifty genes from the C2 gene family were found to be significantly linked to the upregulation of ERBB3 isoforms. The most active gene sets were related to DNA repair, protein secretion, and androgen response, indicating the key biological pathways impacted by ERBB3 isoform expression changes in renal cell carcinoma.

Utilizing CIBERSORTx, the study examined the expression levels of the ERBB3 isoforms uc001sjh.3 and uc001sjl.3 and their correlation with the proportion of immune cells. The findings showed a negative association of uc001sjh.3 with regulatory T cells, activated NK cells, follicular helper T cells, and CD8 T cells. Conversely, a positive relationship was observed with CD4 memory resting T cells, neutrophils, M2 macrophages, resting mast cells, and monocytes. The transcript variant uc001sjl.3 demonstrated opposite trends, with its upregulation leading to a decrease in M2 macrophages, monocytes, and neutrophils, thereby influencing the innate immune response in cancer (Figure 4A). In parallel, the study employed GSEA to assess the gene set enrichment resulting from ERBB3 isoform overexpression, specifically in the context of renal cell carcinoma. The analysis identified significant links between the upregulation of ERBB3 isoforms and fifty genes from the C2 gene family. These genes were primarily involved in DNA repair, protein secretion, and androgen response, as visually represented in a heatmap (Figure 4B).

### 3.4. Expression Changes in Specific Exon Regions of ERBB3

**Figure 5:**
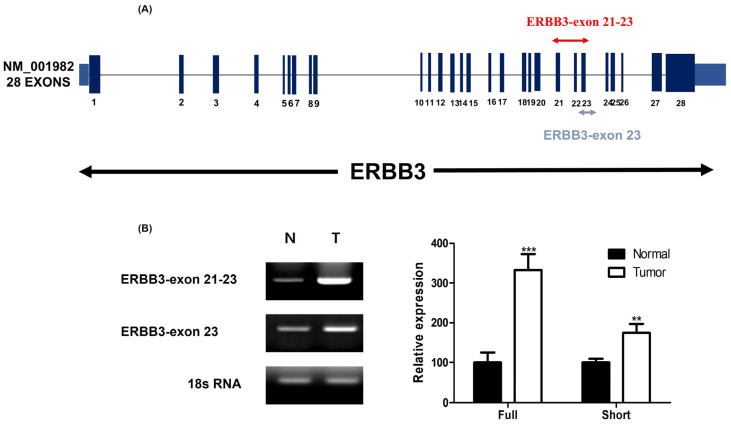
Assessment of ERBB3 expression in specific exon regions of renal cell carcinoma tissues via RT-PCR and quantitative analysis. (**A**) RT-PCR analysis was performed on specific exon regions (exons 21–23 and exon 23) of the ERBB3 gene in both normal and tumor tissues from patients with renal cell carcinoma. We wanted to confirm whether the gene would be expressed even if alternative splicing was performed on a specific region of ERBB3 rather than the entire gene. (**B**) In both areas, the amount of ERBB3 expression was higher in tumor tissue compared to normal tissue. ImageJ software was used to analyze the quantified RT-PCR results for ERBB3 expression in normal tissue and tumor tissue. This confirms that the observed changes in ERBB3 expression between normal and tumor tissues are statistically significant. ** *p* < 0.01, *** *p* < 0.001, indicating a significant difference from the normal group.

Utilizing a range of analytical techniques, we have identified a correlation between patient disease prognosis, survival rates, and the type and expression levels of ERBB3 isoforms. To further validate these findings, we concentrated on the exon 21–23 and exon 23 regions of the ERBB3 gene. We developed primers for these specific regions in both the normal and tumor tissues of patients, opting not to analyze the entire ERBB gene (Figure 5A). Our RT-PCR analysis of these regions showed an increased expression of ERBB3 in tumor tissues compared to normal tissues in both cases. Additionally, we employed ImageJ software to quantify the RT-PCR results, which verified that these variations are statistically significant (Figure 5B). The RT-PCR results for 26 patients are detailed in Figure S3.

## 4. Discussion

ERBB3, a member of the ERBB family of receptor tyrosine kinases, has emerged as a key player in the pathogenesis of various cancers, including renal cell carcinoma (RCC). The role of ERBB3 in cancer biology is complex, as it is involved in cell proliferation, differentiation, and survival. Researching the functions and expressions of ERBB3 isoforms is important for creating targeted therapies and improving patient outcomes in renal cell carcinoma (RCC), a type of cancer that has a very complex genetic landscape. Our study’s detailed examination of ERBB3 isoforms in RCC reveals intricate expression patterns that may be pivotal in understanding the disease’s heterogeneity and complexity. The isoforms uc001sjg.3 and uc001sjh.3 are expressed less in tumor tissues, which suggests that they might play a role in stopping tumors from growing. On the other hand, the fact that isoforms like uc010sqb.2 and uc001sjl.3 are common in tumor samples may mean that they play a role in oncogenic pathways. Understanding the specific roles of different isoforms is important for creating targeted therapies that go beyond generalizations about ERBB3’s function and show how it works in RCC. Our research contributes a new dimension to the existing literature by focusing on isoform-specific expressions in RCC. This approach adds granularity to our understanding of ERBB3’s role in cancer, enabling us to piece together a more comprehensive picture of its multifaceted involvement. The differential expression of these isoforms not only suggests their potential as therapeutic targets but also as prognostic biomarkers, which could improve early detection and patient outcomes. Targeting specific ERBB3 isoforms that are overexpressed in RCC could be a more targeted and effective way to treat the disease, which is in line with the goals of personalized medicine. However, the observational nature of our study precludes definitive conclusions about the causative roles of different ERBB3 isoforms in RCC. The reliance on retrospective data, while comprehensive, may introduce inherent biases. Prospective studies and functional assays are needed to validate our findings and explore the mechanistic roles of these isoforms. Further research should also delve into the interactions of ERBB3 isoforms with other molecular pathways in the tumor microenvironment, potentially unveiling new therapeutic targets and deepening our understanding of RCC pathophysiology. This study underscores the importance of isoform-specific research in the field of cancer. As we dissect the molecular complexity of cancers like RCC, the inadequacy of a one-size-fits-all approach becomes evident. Our findings advocate for a shift toward personalized medicine, where understanding the unique molecular landscape of each patient’s cancer can lead to more targeted and effective treatments. By paving the way for such targeted strategies, our research holds promise for significantly improving the landscape of cancer treatment and patient care in RCC.

## 5. Conclusions

Our findings offer a significant advancement in the understanding of the role of ERBB3 isoforms in RCC, highlighting their multifaceted impact on cancer biology. The study illustrates how these isoforms not only play a role in determining tumor behavior and patient outcomes but also potentially interact with and modulate the immune landscape within the tumor microenvironment. The associations between ERBB3 isoform expression and key oncogenic pathways underscore their potential as biomarkers and therapeutic targets, suggesting a promising avenue for personalized medicine in RCC treatment. This research sets a precedent for future studies aimed at unraveling the full spectrum of ERBB3 isoform functionality, which could revolutionize approaches to RCC treatment and patient care.

## Figures and Tables

**Table 1:** Patients’ clinical characteristics.

NO.	Patient ID	Laterality	Type of Surgery	Histologic Subtype	Tumor Size	Nucleolar Grade (WHO/ISUP)	Tumor Necrosis	Pathologic Stage (AJCC 2017):
#1	KC-9	right	radical nephrectomy	clear cell type	3.5 cm	2/4	0%	pT3aNx
#2	KC-10	right	radical nephrectomy	clear cell type	3 cm	2/4	0%	pT1aNx
#3	KC-11	right	partial nephrectomy	clear cell type	9.1 cm	3/4	10%	PT3bNx
#4	KC-12	left	radical nephrectomy	clear cell type	2 cm	2/4	0%	pT1aNx
#5	KC-13	left	radical nephrectomy	clear cell type	2.8 cm	2/4	0%	pT1aNx
#6	KC-14	left	partial nephrectomy	clear cell type	7.2 cm	3/4	5%	pT2aNx
#7	KC-15	right	partial nephrectomy	clear cell type	8 cm	3/4	10%	pT3aNx
#8	KC-16	right	radical nephrectomy	papillary type 1	1.5 cm	3/4	5%	pT1aNx
#9	KC-17	right	radical nephrectomy	clear cell type	1.8 cm	3/4	10%	pT1aNx
#10	KC-20	left	radical nephrectomy	clear cell type	1.5 cm	3/4	0%	pT1aNx
#11	KC-21	right	radical nephrectomy	clear cell type	1.4 cm	2/4	0%	pT1aNx
#12	KC-22	left	radical nephrectomy	papillary type 2	2.8 cm	3/4	0%	pT1aNx
#13	KC-23	right	partial nephrectomy	clear cell type	2.5 cm	2/4	3%	pT3aNx
#14	KC-24	left	partial nephrectomy	leiomyoma	7 cm	4/4	10%	ypT3a
#15	KC-26	left	partial nephrectomy	clear cell type	2.2 cm	2/4	0%	pT1aNx
#16	KC-27	left	radical nephrectomy	clear cell type	1.1 cm	2/4	0%	pT1aNx
#17	KC-28	left	radical nephrectomy	clear cell type	2.5 cm	3/4	0%	pT1aNx
#18	KC-29	left	radical nephrectomy	clear cell type	4.4 cm	4/4	60%	pT1bNx
#19	KC-32	left	partial nephrectomy	clear cell type	2.2 cm	2/4	10%	pT1aNx
#20	KC-36	right	partial nephrectomy	clear cell type	3.5 cm	4/4	10%	pT1aNx
#21	KC-37	left	radical nephrectomy	clear cell type	7.8 cm	4/4	20%	pT3aNx
#22	KC-38	left	partial nephrectomy	clear cell type	3.5 cm	4/4	10%	pT1aNx
#23	KC-43	left	radical nephrectomy	clear cell type	4.5 cm	3/4	10%	pT1bNx
#24	KC-51	left	partial nephrectomy	clear cell type	3.6 cm	3/4	10%	pT1aNx
#25	KC-52	left	radical nephrectomy	clear cell type	8.8 cm	3/4	10%	pT2aNx
#26	KC-57	right	partial nephrectomy	clear cell type	3.8 cm	3/4	10%	pT3aNx

**Table 2:** RT-PCR primer sequences.

Genes		Sequence (5′-3′)	Annealing Temperature (°C)
18S	Forward Reverse	5’-CTGCCCTATCAACTTTCGATGGTA-3’ 5’-CCGTTTCTCAGGCTCCCTCTC-3’	54 °C
ERBB3 23 Exon	Forward Reverse	5’-TGATGACCTTCGGGGCAGAG-3’ 5’-CATCAATTGTGCAGATCTGGGG-3’	57 °C
ERBB3 21 Exon	Forward	5′-ATGGTGCATAGAAACCTGGCT-3′	55 °C

## Data Availability

Not applicable.

## Supplementary Materials

The following supporting information can be downloaded at: https://www.mdpi.com/article/10.3390/medicina60010181/s1, Figure S1: ERBB isoforms with no significant differences between normal and tumor tissues; Figure S2: Two types of ERBB3 isoforms (uc001sig.3, uc010sqb.2) with no specific correlation in patient survival rate and disease prognosis; Figure S3: RT-PCR result for a specific exon region of ERBB3.
