# Supplementary figures and images for: Deciphering the Role of ERBB3 Isoforms in Renal Cell Carcinoma: A Comprehensive Genomic and Transcriptomic Analysis
Source: Medicina (Kaunas). 2024 Jan 20;60(1):181. doi: 10.3390/medicina60010181 (PMC10820170; doi:10.3390/medicina60010181)

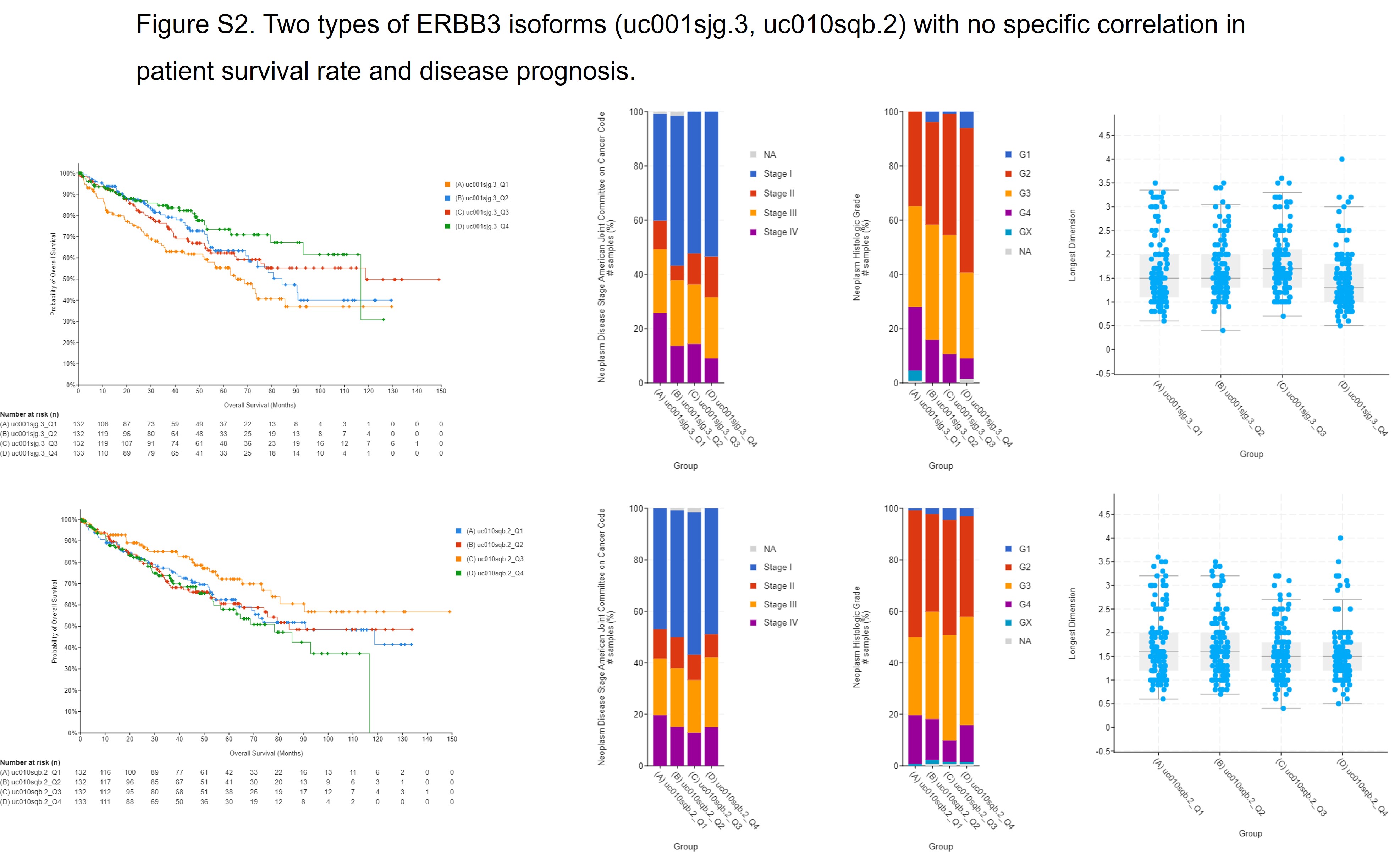

Supplement: Supplementary file 1 [file medicina-60-00181-s001.zip › medicina-2838495-supplementary/Figure S2.jpg]

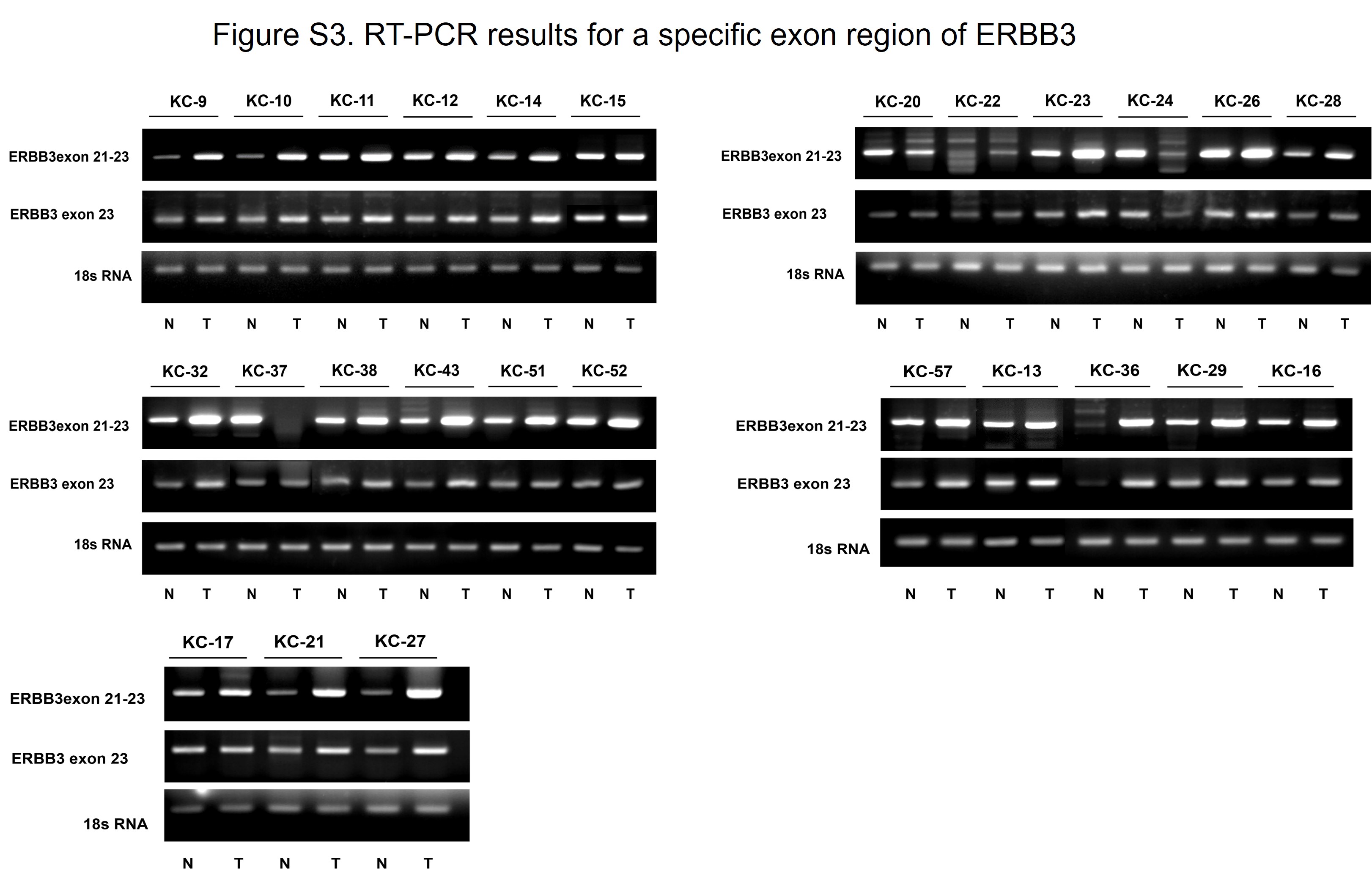

Supplement: Supplementary file 1 [file medicina-60-00181-s001.zip › medicina-2838495-supplementary/Figure S3.jpg]

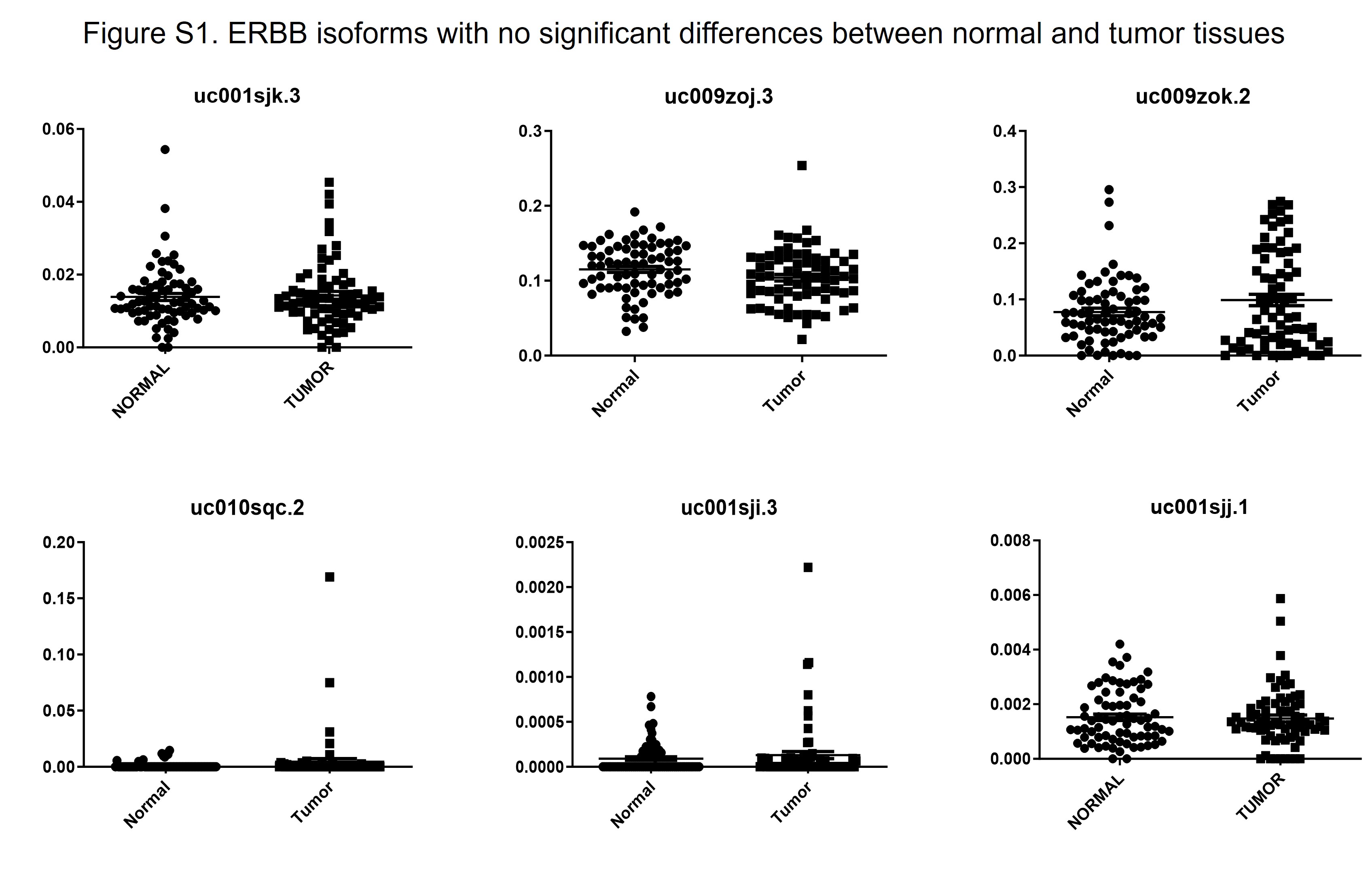

Supplement: Supplementary file 1 [file medicina-60-00181-s001.zip › medicina-2838495-supplementary/Figure S1.jpg]
